# Implementation of a midwifery model of woman-centered care in practice: Impact on oxytocin use and childbirth experiences

**DOI:** 10.18332/ejm/146084

**Published:** 2022-04-01

**Authors:** Ingela Lundgren, Anna Dencker, Marie Berg, Christina Nilsson, Liselotte Bergqvist, Ólöf-Ásta Ólafsdóttir

**Affiliations:** 1 Institute of Health and Care Sciences, Sahlgrenska Academy, University of Gothenburg, Gothenburg, Sweden; 2 Sahlgrenska University Hospital, Gothenburg, Sweden; 3 Munkebäck Antenatal Clinic, Gothenburg, Sweden; 4 Department of Midwifery, Faculty of Nursing, University of Iceland, Reykjavík, Iceland

**Keywords:** midwifery, mixed method, woman-centered care, theoretical models of care

## Abstract

**INTRODUCTION:**

Theoretical models for midwifery have been developed in different countries, but few have been evaluated. This study evaluated the implementation of a midwifery model of woman-centered care (MiMo) in practice.

**METHODS:**

A mixed method study based on an implementation of MiMo was carried out in a labor ward at a university hospital in Sweden, with another labor ward as a reference. The qualitative core component was a secondary analysis of focus groups with midwives after the implementation. The supplemental quantitative components were oxytocin use for augmentation of labor and women’s childbirth experiences before and after the implementation.

**RESULTS:**

The midwives viewed MiMo as a useful tool for comprehending the birthing woman holistically, and for identifying what might disturb the birth process. Hindering factors were a lack of organizational stability and time, and midwives’ unwillingness to understand the model. Oxytocin use decreased significantly only in the implementation ward (p=0.002) and a significant difference was found between wards in the post-implementation period (p=0.004). However, logistic regression analyses showed that the interaction between ward and time period, controlling for age, epidural use, and birth outcome, was not significant (p=0.304), indicating that the decrease was not significantly related to the implementation. Childbirth experience did not differ before and after the implementation.

**CONCLUSIONS:**

By using MiMo in practice, midwives have a tool for comprehending the woman holistically and identifying disturbing factors during the birth. However, more research is needed for further implementation that should focus on the potential as well as hindering factors.

## INTRODUCTION

Theoretical models of midwifery care have been developed over the last decades. A recent scoping review identified six models, developed in the US^1^, New Zealand and Scotland^2^, South Africa^3^, Sweden^4,5^, and Iceland^5,6^. These models differ in their philosophical underpinnings and methodologies, but they have in common a distinctive conceptualization of midwives’ relationships with women, a focus on woman-centeredness, and a salutogenic approach^7^.

Few theoretical models have been validated in terms of usefulness in practice. Two models from the US^1,8^ conclude that the models give structure for future research on the unique aspects of midwifery care^1^, showing that the concept of positive presence in nurse-midwifery care for women enhances satisfaction with the birth experience^8^. Most of the models are from the UK, the US, and New Zealand, involving private care, continuity of care, informed choice for women, and different professional responsibility of midwives relative to those of obstetricians^1,2,8-13^. Since the cultural context of maternity care and professional responsibility differs between countries, there was a need for a model based on the Nordic context where midwives have similar long history; the first midwives were educated in the beginning of the 1700s, having an independent responsibility for normal births and obstetricians for complicated births^14^. Today, most women give birth in hospital-based obstetric-led labor wards with midwives attending the births, and there are very few alternatives for women, such as midwife-led birth centers, continuity models of care, and home births. The Nordic countries have low cesarian section (CS) rates and among the lowest rates of maternal and infant mortality^15^.

A midwifery model of woman-centered care (MiMo) was developed by a hermeneutic secondary synthesis of 12 qualitative study findings on either women’s or midwives’ experiences of childbirth and validated in focus groups by midwives in Sweden and Iceland. In this model, the concept of ‘midwifery’ is used for midwives’ praxis in maternity care, and the concept of ‘woman-centered care’ means that the care is based on women’s views and needs in childbirth^5^. An advanced concept analysis about woman-centered care^16^ based on five studies from New Zealand, one from the UK, and one from Australia confirms that most studies are carried out in a context similar to the one mentioned above. One study in the analysis was from Sweden and Iceland (MiMo). The findings show that woman-centered care has both a philosophical and a pragmatic meaning, and a dual and equal focus on the woman’s individual experience, meaning, and manageability of childbearing and childbirth, and on the health and well-being of mother and child.

The study presented here is part of a research project with the overall aim of evaluating the usefulness of MiMo and developing guidelines for its use in practice. Four studies have been published after the MiMo implementation. One study shows that MiMo is useful for midwives, managers, and obstetricians, as it gives words to midwifery care, but there is a need to clarify professional roles and interdisciplinary collaborations for further developments^17^. The participating hospital has a ‘baby factory context’, where midwives’ care was perceived as ‘veiled’ by other professions working in the same ward. This generates a desire to streamline and control midwifery in order to increase interprofessional collaboration^18^. Further, the midwives work in a field of tension and have to balance contrasting models of care, which indicates the need for a model but can also hinder the implementation of a model^19^. Another study shows that MiMo has the potential to strengthen the professional role and midwifery practice but not the strained work situation for midwives^20^.

A reciprocal relationship and supporting normality in a birthing atmosphere are central themes in MiMo^5^. Negative and traumatic birth experiences are often related to lack of support during the birth, with a negative impact on the woman’s health and well-being and her relationship with her child^21,22^. Continuous support during birth is related to an improved birth experience and less intervention and more normal births for women^23^. The idea was that the use of MiMo with a focus on the reciprocal relationship between the midwife and the woman in a birthing atmosphere that supports normality improves women’s birth experiences and minimizes interventions such as oxytocin use in the birth process. The aim was to explore and analyze MiMo in relation to the use of oxytocin for augmentation of labor and to women’s childbirth experiences.

## METHODS

### Study design and setting

This study as well as the MiMo research project have a mixed method design^24^ using both quantitative and qualitative research data before and after implementation. The MiMo research project included a one-year implementation of MiMo at a university hospital in the western part of Sweden. Two wards were included in the study; one was used for the implementation and one was situated in another part of the city as a reference ward. The participating hospital has also a high-risk ward for women with high risk during pregnancy not involved in this study. Data were gathered before and after the implementation. More in-depth information about the MiMo implementation, the different professionals’ perspectives, and the context have been published^17-20^.

The core components in this mixed method study^24^ was a secondary analysis^25^ of data from focus-group interviews with midwives. Two questions were asked in the original focus groups before and after the implementation: ‘what is your opinion of the practical usefulness of midwives adopting MiMo’, and ‘what is your professional role related to woman-centered care?’^17^. In this study, the focus groups were reanalyzed focusing on midwives’ views of MiMo related to the use of oxytocin and women’s childbirth experiences and after the implementation. The supplemental component was an analysis of two quantitative measures: whether the use of MiMo was associated with decreased use of oxytocin for augmentation of labor and whether it showed improvement of women’s childbirth experience as measured using the Childbirth Experience Questionnaire (CEQ2)^26^. Finally, the qualitative core findings and quantitative supplemental findings were synthesized by contrasting and corresponding the core findings with the supplemental findings^24^.

The implementation ward (IW) and the reference ward (RW) were mainly for normal birth, i.e. women with singleton uncomplicated pregnancies and expected uncomplicated births from gestational week 34+0. However, induction of labor was common, as were cases where women had minor complications such as gestational diabetes and gestational hypertension during pregnancy. Midwives in Sweden have an independent responsibility for normal births^14^. When complications occur, an obstetrician takes over the responsibility, but the midwives remain involved in the woman’s care.

Approximately 80 midwives were employed at the IW and 80 at the RW. Continuous support was not offered to all women since the midwives often had to care for more than one woman during labor and birth at IW and RW.

**Figure 1:**
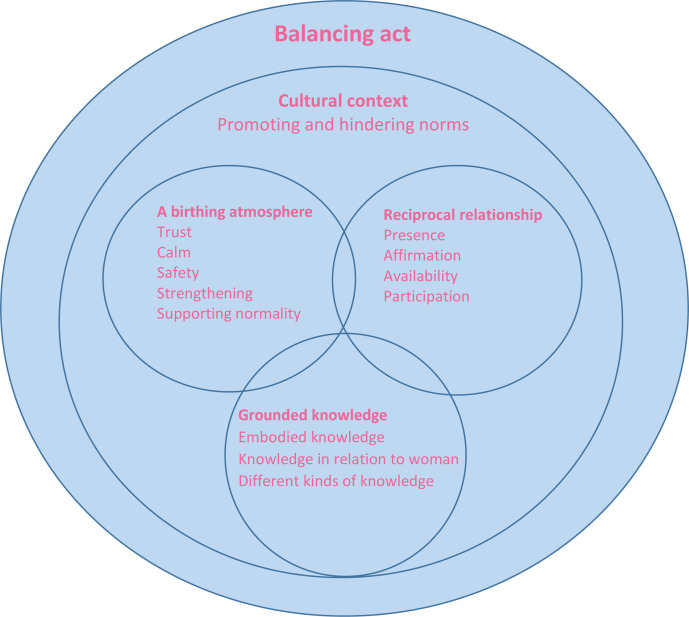
The midwifery model of woman-centered care (MiMo)

The MiMo implementation comprised three parts:

1. One education day (8 hours) for all midwives, with handouts about the model, including a MiMo card (Figure 1) with the model and logo of the MiMo research project to be used for guiding the implementation on the ward. The researchers (IL, MB, CN, OAO) presented the different parts of the model, and group discussions were conducted based on questions in relation to the model and on participants’ own questions; 75 midwives participated.
2. One-hour meetings for the introduction of the model to each group of obstetricians, assistant nurses, and managers.
3. Regular reflection group meetings with midwives throughout the study period, i.e. six occasions/midwife × 1.5 hours. The purpose of the reflection groups was to deepen the midwives’ understanding of MiMo. In the meetings, they reflected on actual cases in relation to the themes of MiMo. The reflection groups were principally led by one of the researchers (CN), however, four ‘MiMo midwives’ who showed particular interest in the research were recruited as reflection group leaders. They were trained and supervised by CN and had two additional educational hours with the MiMo research team.

During the implementation, March 2015–March 2016, the midwives at the IW were free to use the model in their daily practice, but they were not obliged to. No routines or guidelines were changed at the IW. The care at the RW was not changed during the study period. The participating hospital had one ongoing quality project^27^ in all wards (including the high-risk ward) during the MiMo implementation, with the goal of improved quality of care in childbirth for primiparous women. The quality project involved monthly meetings with some of the professionals (midwives, obstetricians, and assistant nurses) at which the care was discussed based on monthly statistics about and the rates of CS, anal sphincter tears, and augmentation of oxytocin. However, no specific education was carried out during the MiMo implementation.

### Participants, data collection and data analysis

To describe midwives’ views on the use of MiMo in relation to the use of oxytocin for augmentation and childbirth experiences, the transcripts of two focus group discussions with a total of 11 midwives working at the IW were reanalyzed with inductive content analysis^25^, by IL, CN and OAO. In the new secondary analysis, units of analysis were marked which answered the research questions. These units of analysis were read to get a sense of the data as a whole, followed by open coding using coding sheets, and then grouping, categorizing, and abstracting the data. The midwives gave informed consent before participating in the focus groups.

For measuring the use of oxytocin for augmentation of labor, all primiparous women giving birth before (1 January 2014 – 28 February 2015) and after the MiMo implementation (1 April 2016 – 30 April 2017) at IW and RW, were included. Exclusion criteria were induction of labor and elective CS. The study population for the oxytocin augmentation outcome comprised 6882 primiparous women, of which 1813 women were included at the IW, and 1774 at the RW before the implementation. After the implementation, 1697 women were included at the IW and 1598 women at the RW.

For measuring childbirth experiences, primiparous women were included before (1 January – 24 August 2015), and after the implementation (9 March – 1 December 2016). The women were recruited when they came to a routine check-up for the baby at the hospital two to three days after the birth, and those who agreed to participate left their contact email address. The web questionnaire, including 22 items from CEQ2^26^ and a few background questions, was emailed 3–4 weeks postpartum. Two reminders were sent. Exclusion criteria were induction of labor and elective CS. The study population for childbirth experience comprised 810 primiparous women, of which 173 were included at the IW, and 132 at the RW before the intervention, and 250 women were included at the IW and 255 women at the RW after the intervention.

Information about augmentation with oxytocin in primiparous women (yes/no) was collected from Obstetrix (electronic record system for all births at the participating hospital). Total scores on CEQ2 ranged 1–4, where a high score corresponds to a good experience.

Sample size was calculated to detect a 10% difference between wards in augmentation use in primiparous women with spontaneous onset of labor. A total of 1600 primiparous women per group pre- and post-implementation were estimated to achieve 80% power at a 5% level of significance.

Descriptive statistics were used to characterize the study sample and results. Differences in oxytocin use (yes/no), birth outcome (vaginal non-instrumental/instrumental) and epidural use (yes/no) were evaluated separately between wards for Period 1 and Period 2 and within wards between periods using the χ^2^ statistic. Differences in maternal age were calculated with t-tests. Logistic regression was used to examine the effects of the implementation on oxytocin use. An initial model with period (1, 2), ward (RW, IW), birth outcome, epidural use and maternal age entered simultaneously, was examined for significant candidates for the next model. A second model included an interaction term between period and ward, controlling for significant independent predictors from the first. Reference categories were designated Period 1 and RW for interpreting odds ratios for the variables in the interaction term. Overall model goodness-of-fit was evaluated with the Hosmer and Lemeshow Test and percentage of variance with the Nagelkerke pseudo R^2^. Total scores on the CEQ2 were compared using the Mann-Whitney U-test. Statistical significance was set to p<0.05 throughout. All analyses were performed using IBM Statistics version 24 (IBM Corp: New York, USA).

## RESULTS

### Qualitative core component

Four main categories describe midwives’ views concerning the use of oxytocin and childbirth experiences.

*A tool for extended understanding of what may disturb the woman during labor and birth*

MiMo was considered to be a tool that could help the midwives notice disturbance around the woman, influencing her birthing process. By using MiMo, it was easier to conceptualize dimensions that hinder the woman from focusing on giving birth. The midwives looked at the different circles on the MiMo card, about the birthing atmosphere, grounded knowledge, and a reciprocal relationship, which helped them figure out the many things that are involved:

*‘When you look at the model, it's easier to figure out the problem. It's not only about progress of labor, it's more complex, not only the partogram. When you look at the model you realize how many things have an influence.’*

Oxytocin for augmentation is often used as the only solution for prolonged labor. Sometimes midwives use oxytocin even without considering the reasons for prolonged labor. According to the midwives, the rate of oxytocin use is too high in the ward. By using MiMo, they were motivated to think in another way and have other solutions, such as focusing more on the reciprocal relationship, being present at the side of the woman, and maintaining a calm birthing atmosphere:

*‘When it's prolonged labor we often immediately administer a drip without questioning why it is a prolonged labor? When using MiMo you have to think in another way.’*

In the ward they use time-out after 2–3 hours of prolonged labor, which means that the team around the woman gathers for a discussion about solutions. The woman should also be involved, and for the midwives, MiMo was a tool they could use for these discussions. They could use the MiMo card themselves and show it to the team. However, the other professions sometimes had problems comprehending the model.

Fear of giving birth is one disturbing dimension, according to the midwives. Then the woman may have problems with entering into and following the birth process, with a risk of prolonged labor. Supporting women with fear of giving birth is one aspect that should be conceptualized in the model.

*A tool that helps midwives comprehend the birthing woman holistically*

A holistic approach is of importance when trying to understand the woman and help her achieve a positive birth experience. MiMo was seen as a tool that could help the midwives enter more deeply into the encounter with the woman and her partner. The midwives also reflected on their own role in the encounter with the woman. Some midwives used the model when teaching midwifery students about comprehending the birthing woman holistically:

*‘I think you can use the model even before problems may arise. I have used it when I talked to a midwifery student about taking a holistic approach to the birth.’*

MiMo could be a tool for all situations and used during medical rounds for discussions about how the woman may achieve a normal birth and not mainly focus on problems during the birth, such as when the birth process is prolonged:

*‘I think that you can use this (points at MiMo) in all situations; even if it's a prolonged labor, you can reflect more deeply on why this situation has happened. However, it is demanding and requires work experiences as a midwife.’*

*A tool that requires a willingness to understand*

MiMo requires willingness to understand how to use it, looking at childbirth in wholeness. On their busy labor ward, the midwives faced many norms that hindered them from fully using this theoretical model of care. At the beginning of the implementation period, midwives expressed that they had previously and intuitively worked in the dimensions MiMo describes. However, at the same time, some of the midwives expressed that MiMo was hard to understand and that they had to make a point of using it, knowing that it could be helpful to reflect on their work:

*‘This is rather different; it takes time to enter into these questions. You need to get past obstacles.’*

Therefore, reflection groups were of importance for understanding the model. Reflection groups were also an important tool that the midwives asked for before the MiMo implementation. Because of their heavy workload, it was sometimes hard for the midwives to prioritize the reflection groups’ meetings even if they were scheduled.

In relation to willingness to understand, in some situations it was hard for the midwives to relate their cases to the model. Then the model was too much the main issue rather than their own reflections. Further, sometimes it was hard for the midwives to give constructive critiques to others, such as whether the use of medical interventions was necessary; instead, they just confirmed that they would have done the same.

*Stability and time are needed for building up a relationship*

Lack of time hindered the midwives from entering more deeply into the encounter and developing a relationship with the women and thereby understanding what may disturb the women and the birth process. They expressed that there was often too little time available for helping the woman achieve a good birth experience:

*‘It is not right to ruin the woman's birth experience just because it's a busy day on the ward.’*

Further, stability is needed in the organization for using the model in practice. The midwives needed support from the organizational management. Often the midwives have too many tasks during their workday that hinder them from focusing on the guidance included in the dimensions in MiMo and thereby from building up a relationship with the individual woman. Also, the midwives themselves needed time and support from the organization in order to give each woman the support she needed.

### Quantitative supplemental component

Oxytocin use, birth outcome, epidural use and maternal age for primiparous women with spontaneous onset of labor at IW and RW during Periods 1 and 2 are presented in Table 1.

**Table 1:** Birth outcome and interventions during the birth for primiparous women with spontaneous onset of labor (N=6882)

*Characteristics*	*Implementation ward (n=3510; 51%) n (%)*		*Reference ward (n=3372; 49%) n (%)*	
	*Period 1^a^*	*Period 2^b^*	*Period 1^a^*	*Period 2^b^*
**Births**	1813 (51.7)	1697 (48.3)	1774 (52.6)	1598 (47.4)
**Age** (years), mean ± SD	29 ± 5.01	29 ± 4.90	29 ± 4.58	29 ± 4.60
**Birth outcome**				
Vaginal, non-instrumental	1477 (81.5)	1439 (84.8)	1499 (84.5)	1363 (85.3)
CS	168 (9.3)	127 (7.5)	147 (8.3)	128 (8.0)
Vacuum and forceps	168 (9.3)	131 (7.7)	128 (7.2)	107 (6.7)
**Epidural**	872 (48.1)	776 (45.7)	762 (43.0)	708 (43.6)
**Augmentation with oxytocin**	877 (48.4)	731 (43.1)	876 (49.4)	769 (48.1)

a Before the intervention: 1 January 2014 – 28 February 2015.

b After the intervention: 1 April 2016 – 30 April 2017.

**Figure 2:**
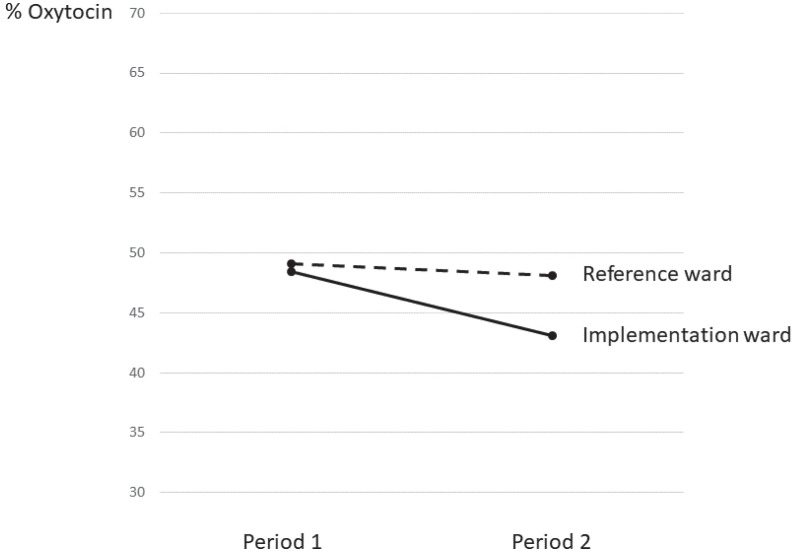
Rates of oxytocin stimulation between the Implementation Ward and the Reference Ward

In Period 1, no significant difference in rates of oxytocin stimulation was found between the IW (48.4%) and RW (49.4%), (Figure 2). In Period 2, oxytocin use was lower in both wards (IW=43.1%, relative difference= -11%; RW=48.1%, relative difference= -3%); however, the difference between periods was significant only in IW (p=0.002). IW also had significantly lower oxytocin use than RW during Period 2 (p=0.004). Regarding the other study variables, significantly higher rates of epidural use (p=0.002) and instrumental births (p=0.016) were observed in IW during Period 1. Instrumental births decreased significantly only in IW (p=0.009)

A logistic regression analysis without an interaction term revealed that ward, period, birth outcome, epidural use and maternal age were all significant independent predictors of oxytocin stimulation (Nagelkerke pseudo R^2^=0.30; χ^2^=18.17, df=8, p=0.02; overall classification=72.2%); however, a subsequent model showed that the interaction between ward and time period, controlling for birth outcome, epidural use and maternal age, was not significant, indicating that the decrease in oxytocin use was not significantly related to the implementation (Table 2) (Nagelkerke pseudo R^2^=0.30; χ^2^=19.47, df=8, p=0.013; overall classification=72.2%).

**Table 2:** Logistic regression analysis of factors affecting oxytocin use for augmentation of labor in primiparous women with spontaneous onset of labor

*Variable*	*b*	*S.E.*	*Wald*	*df*	*Sig*	*Exp(b)*	*95% CI for Exp(b)*	
							*Lower*	*Upper*
Ward^a^	-0.195	0.077	6.458	1	0.011	0.823	0.708	0.956
Period^a^	-0.082	0.079	1.071	1	0.301	0.922	0.789	1.076
Period by ward	-0.114	0.111	1.058	1	0.304	0.892	0.718	1.109
Epidural	1.886	0.056	1131.766	1	0.001	6.594	5.907	7.359
Birth outcome	-1.190	0.082	208.252	1	0.001	0.304	0.259	0.358
Maternal age (years)	0.054	0.006	79.466	1	0.001	1.055	1.043	1.068
Constant	-1.365	0.206	43.896	1	0.001	0.255		

a Reference categories: Ward: reference ward. Period: period 1.

At the IW, the mean total score for childbirth experience was 3.27 before and 3.36 after the intervention (p=0.088). In the RW, mean scores were 3.19 and 3.27, respectively (p=0.088) (Table 3).

**Table 3:** Total score of childbirth experience

	*Hospital*							
	*Intervention ward*				*Reference ward*			
	*Total*	*Period 1^a^*	*Period 2^b^*	*pc*	*Total*	*Period 1^a^*	*Period 2^b^*	*pc*
Primiparous women with spontaneous onset of labor	423	173	250		387	132	255	
Level of total CEQ score, mean ± SD (median)	3.32 ± 0.49 (3.46)	3.27 ± 0.53 (3.45)	3.36 ± 0.47 (3.48)	0.88	3.24 ± 0.52 (3.35)	3.19 ± 0.53 (3.29)	3.27 ± 0.51 (3.39)	0.88

a Before the intervention: 1 January – 24 August 2015.

b After the intervention: 9 March – 1 December 2016.

c Mann-Whitney U-test.

### Synthesized findings

The qualitative findings showed that MiMo was a useful tool for midwives for exploring and analyzing what disturbs the process of birth, helping them understand the birthing woman holistically and enhance positive birth experiences. However, the quantitative findings showing that women’s childbirth experience did not differ before and after the intervention do not support the qualitative findings. One explanation based on the qualitative findings could be that stability and support from organizational management are lacking. Lack of time and thereby individual support to women was expressed as hindering the midwives from helping the women achieve a good childbirth experience. The qualitative findings further shows that MiMo influenced the midwives to enter more deeply into the encounter and develop a relationship with the woman to support her individual needs. Thereby the midwives had other strategies than oxytocin to prevent prolonged labor and to achieve a normal birth. These qualitative findings are supported by the quantitative that showed a significant decrease in oxytocin use in the IW but not in the RW. Further the quantitative findings showed a decreased rate of instrumental births at the IW. However, the interaction between ward and time period, controlling for age, epidural use and birth outcome, was not significant, indicating that the decrease was not significantly related to the MiMo implementation. One explanation could be lack of time, and support from the organization that hindered midwives from understanding and using MiMo in practice. In summary, the synthesized findings indicate that MiMo may have a potential for midwives work in practice to promote normal births but more studies are needed.

## DISCUSSION

The synthesized results from this mixed method study indicate that, by using MiMo, midwives have a tool for extended understanding of the woman holistically and what may disturb the woman during labor and birth, thereby prompting them to use strategies other than oxytocin for augmentation of labor. However, inadequate stability and time and a lack of willingness to understand are hindering factors for the use of MiMo. The findings from this study are in line with those of other studies in the MiMo research project that show a strained work situation as a hindering factor^19,20^, but that also promote aspects of MiMo that give words to midwifery^17^, to midwives’ professional role, and midwifery practice^20^. The findings from this study add knowledge about how midwives can use MiMo in practice for caring for the woman holistically and about what might disturb the birth process.

The idea behind this mixed method study was that the midwives’ use of the MiMo with a focus on a reciprocal relationship, a birthing atmosphere, and grounded knowledge would improve women’s childbirth experiences and minimize medical interventions. Earlier research shows that continuous support during birth and midwife-led care are related to improved birth outcomes, fewer interventions during birth, and improved satisfaction with the care^23,28^. Further, in a social midwifery model of care, birth is viewed as an event that should not unnecessarily be disturbed^29-31^. However, our study was carried out in a setting where continuity of care, the social model of care, and midwife-led care, are not part of the culture, which may explain the non-significant results. To our knowledge, this is the first study where a midwifery model of woman-centered care has been tested in a big university hospital ward. We have found only one theoretical model that has been tested in practice, Lehrman’s model at a birth center in the US^8^ showing that the concept of positive presence in nurse-midwifery care for women enhances satisfaction with the birth experience.

According to our findings, the use of MiMo has potential in a hospital-based labor ward but can also been used in other settings. The studies involved in the development of MiMo were from hospital births as well as birth centers and home births^5^. MiMo can be used as a tool for midwives’ care to promote woman-centered care and use midwifery skills in all childbirth care settings as well as in midwifery education. However, since most women give birth in hospitals, there is a need to develop and test woman-centered care models in these settings. Further woman-centered care models should be available for all women, not only midwife-led care, continuity of care models, and in normal births^32^.

This study had women’s childbirth experiences as one quantitative outcome with no apparent change after the intervention. This is in contrast to how other woman-centered models of care and continuous support are related to more satisfaction with the care^23,28^. Lehrman’s model^8^ also found a contribution to women’s satisfaction with their labor and birth experience where positive presence from caregivers was central. During the implementation period, the midwives expressed and showed willingness to create opportunities to focus on MiMo. However, they also described how lack of time was a factor that hindered them from using MiMo and helping women achieve a positive childbirth experience. Further, they explained that stability and willingness to understand this complex theoretical model in depth was important for using MiMo in practice, which may be a reason for the non-significant findings.

The synthesized findings indicate that MiMo may be tool for understanding and using strategies other than oxytocin for prolonged labor and to achieve a normal birth. From 2013 to 2017, the participating hospitals statistics based on the quality project^27^ show improved birth outcomes; the emergency CS rate decreased from 9.6% to 7.0%, anal sphincter tears from 5.5% to 3.5%, and augmentation with oxytocin from 50% to 48%. In 2017, the year after the MiMo study, the rate of augmentation with oxytocin increased in the implementation ward to 48%, and a similar rate existed in 2020, compared to 43.1% after the MiMo implementation in 2015 (personal communication, Liselotte Bergqvist), which indicates that MiMo may have had an impact on oxytocin use. It is also in accordance with the qualitative component of findings about how midwives thought that MiMo could be used to prevent prolonged labor. More controlled studies are needed to further assess MiMo’s relative merits in reducing medical interventions and to explore its impact on outcomes of childbirth care.

## CONCLUSIONS

The synthesized findings from this mixed method study indicate that, by using MiMo in practice, midwives have a tool for understanding the woman holistically and for identifying what may disturb the woman during the birth and thereby have strategies other than oxytocin for prolonged labor. Hindering factors for the use of MiMo are a lack of willingness to understand, inadequate support from management, and a lack of stability and time, which also may be the reasons for the non-significant quantitative findings of the study. Further research is needed to explore the impact of MiMo and developing its use in practice.

## Data Availability

The data supporting this research cannot be made available for privacy reasons and due to ethical guidelines concerning qualitative interviews.
